# The Role of Ferroptosis in the Treatment and Drug Resistance of Hepatocellular Carcinoma

**DOI:** 10.3389/fcell.2022.845232

**Published:** 2022-03-03

**Authors:** Siqi Zhao, Wubin Zheng, Chao Yu, Gaoxin Xu, Xinyi Zhang, Chao Pan, Yongheng Feng, Kunxing Yang, Jin Zhou, Yong Ma

**Affiliations:** Department of General Surgery, Nanjing First Hospital, Nanjing Medical University, Nanjing, China

**Keywords:** ferroptosis, drug resistance, treatment, hepatocellular carcinoma, regulatory cell death

## Abstract

Cell death is a fundamental feature of multicellular organisms’ development and a key driver of degenerative diseases. Ferroptosis is a new regulatory cell death mediated by iron-dependent lipid peroxidation, which is different from apoptosis and necrosis in morphology, pathophysiology and mechanism. Recent studies have found that ferroptosis is involved in the development of many diseases including hepatocellular carcinoma (HCC). As further research progresses, specific mechanisms of ferroptosis in HCC are being revealed. In this review, we summarize these recent advances about the treatment of drug-resistance in HCC and the latest ferroptosis-related treatment for HCC.

## Introduction

HCC is an invasive cancer prevalent worldwide, with a mortality rate ranked second among all the cancers, which was just behind lung cancer and colon cancer (Bray et al., 2018). The 5-years survival rate of HCC patients is less than 10%, and the average life expectancy is only 6 months for those patients who were not eligible for surgery. And the existing treatments, including radiofrequency therapy, radiotherapy therapy, and chemotherapy, do not significantly improve the prognosis of HCC patients. Currently, in terms of HCC chemotherapy, the US Food and Drug Administration (FDA) has approved a variety of small molecule multi-kinase inhibitors, such as sorafenib, for the treatment of advanced HCC (Boland and WU, 2018). However, the therapeutic effect of most patients is still limited due to the frequent drug resistance of those inhibitors. Therefore, different modulation strategies and administration routes have been proposed to enhance the antitumor activity of these agents.

Dixon identified an iron-dependent form of cell death in 2012 and defined this modality as ferroptosis. It is now considered that ferroptosis is triggered by both exogenous and endogenous pathways, either by inhibition of cell membrane transporters (cystine/glutamate transporter system) or by activation of iron transporters, serum transferrin, and lactoferrin. Endogenous pathways are activated by blocking intracellular antioxidant enzymes such as glutathione peroxidase 4 (GPX4) (Tang and KROEMER, 2020). Unlike other known modes of cell death, such as apoptosis, necrosis, and autophagy, ferroptosis has unique morphological, biochemical, and genetic characteristics, such as mitochondrial atrophy, increased membrane density, iron, and ROS accumulation.

Recent studies have found that ferroptosis is involved in the proliferation, invasion, and migration of HCC cells, and is also closely related to drug-resistance in HCC, of which the specific mechanism is being gradually revealed.

## Regulation of Ferroptosis in HCC

**FIGURE 1:**
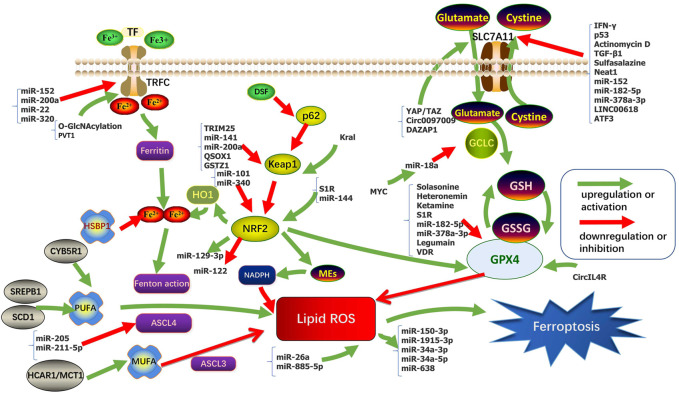
Regulation pathways and key molecular mechanisms of ferroptosis in HCC.

Sensitivity to ferroptosis is closely related to many biological processes, such as (anti-)oxidant metabolism, iron metabolism, lipid metabolism, energy metabolism, and regulation of non-coding RNAs (ncRNAs). NcRNAs participate in the regulation of tumorigenesis *via* various biological processes such as chromatin modification, alternative splicing, competition with endogenous RNAs, and interaction with proteins. Intervention in these key links may regulate the sensitivity of HCC cells to ferroptosis. The regulation of ferroptosis found in HCC in recent years was sorted out in Table 1 and Figure 1.

**TABLE 1:** The regulators of ferroptosis in HCC.

Gene/Axis/Compound/Drug	Mechanism	Target	Influence to ferroptosis	References
Ubiquitin-like Modifier Enzyme 1 (UBA1)	Inhibit NRF2 expression by inhibiting of UBA1	NRF2	-	Shan et al. (2020)
Disulfiram (DSF)	DSF inhibits the signaling pathways of NRF2 and MAPK kinase	NRF2	+	Ren et al. (20211021)
p62	p62 can down-regulate Keap1 expression and reduce NRF2 degradation	Keap1	-	Sun et al. (2016a)
Xanthine Oxidoreductase (XOR)	XOR can down-regulate NRF2 expression	Keap1	+	Sun et al. (2020)
Tripartite motif-containing 25 (TRIM25)	TRIM25 can activate NRF2	Keap1	-	Liu et al. (2020)
Malic enzymes (ME)	Transcriptionally activating ME1 by NRF2 when cells encounter further episodes of ROS insult	induced by NRF2		Lee et al. (2021)
Sigma-1 receptor (S1R)	S1R can regulate NRF2 thus inhibiting ROS accumulation	NRF2	-	Bai et al. (2019)
Catenin beta-1 (CTNNB1)	CTNNB1 may have synergistic effect with NRF2 mutation	NRF2	Unknown	Zavattari et al. (2015); Tao et al. (2021)
miR-101 (miRNA)	Target the 3′-UTR of NRF2 and negatively regulate NRF2	NRF2	+	Gao et al. (2017); Raghunath et al. (2018)
miR-144 (miRNA)	Activation of Nrf2	NRF2	-	Raghunath et al. (2018)
miR-340 (miRNA)	Target at the 3′-UTR of NRF2 and negatively regulate NRF2	NRF2	+	Shi et al. (2014); Raghunath et al., 2018)
miR-122 (miRNA)	Inhibited by NRF2	Inhibited by NRF2	Unknown	Aydin et al. (2019)
miR-129-3p (miRNA)	Induced by NRF2	Induced by NRF2	Unknown	Sun et al. (2019)
miR-141 (miRNA)	Upregulate NRF2	Keap1	-	Raghunath et al. (2018)
miR-200a (miRNA)	Increase NRF2 and inhibit TFR1 expression	Keap1	-	Greene et al. (2013); Raghunath et al. (2018)
Kral (lncRNA)	Induce Keap1 to regulate NRF2	Keap1	+	Wu et al. (2018)
Glutathione S-transferase zeta 1 (GSTZ1)	Inhibit NRF2/GPX4 axis	NRF2	+	Wang et al. (2021a)
Quiescin sulfhydryl oxidase 1 (QSOX1)	Inhibit NRF2	NRF2	+	Sun et al. (2021)
miR-200b (miRNA)	Adjust ferritin heavy chain 1(FtH1) and ferritin light chain (FtL)	Ferritin	Unknown	Greene et al. (2013)
miR-122 (miRNA)	Reduce iron by adjusting Nocturnin	Nocturnin	Unknown	Zhang et al. (2020)
PVT1 (lncRNA)	Increase lipid peroxidation and iron deposition *in vivo* and *in vitro*	TFR1	+	Lu et al. (2020)
miR-152 (miRNA)	Inhibit TFR1 expression	TFR1	-	Kindrat et al. (2016)
miR-22 (miRNA)	Inhibit TFR1 expression	TFR1	-	Greene et al. (2013)
miR-320 (miRNA)	Inhibit TFR1 expression	TFR1	-	Greene et al. (2013)
miR-107 (miRNA)		Inhibited by iron		Zou et al. (2016)
miR-30d (miRNA)		Inhibited by iron		Zou et al. (2016)
Formosaanin C	Inducing ferritinophagy and lipid ROS formation	/	+	Lin et al. (2020)
CDGSH iron sulfur domain2 (CISD2)	Excessive iron ion accumulation	Fe	-	Li et al. (2021b)
O-GlcNAcylation	Increase the iron concentration through transcriptional elevation of TFRC	TRFC	+	Zhu et al. (2021)
Solasonine	Increase lipid ROS levels by suppression of GPX4 and GSS	GPX4	+	Jin et al. (2020)
Heteronemin	Decrease GPX4 expression and induced the formation of ROS	GPX4	+	Chang et al. (2021)
Selenoproteins	Constitute GPX4	GPX4	-	Ingold et al. (2018)
Sigma-1 receptor (S1R)	Inhibit the expression of GPX4	GPX4	-	Bai et al. (2019)
Circ-interleukin-4 receptor (CircIL4R)	As a miR-541-3p sponge to regulate its target GPX4	GPX4	-	Xu et al. (2020)
Ketamine	Decrease expression of lncPVT1 (directly interacted with miR-214-3p to impede its role as a sponge of GPX4) and GPX4	GPX4	+	He et al. (2021)
Legumain	Promote chaperone-mediated autophagy of GPX4	GPX4	+	Chen et al. (2021)
vitamin D receptor (VDR)	Transregulation of GPX4	GPX4	-	Hu et al. (2020)
Ceruloplasmin (CP)	Accumulation of intracellular ferrous iron (Fe^2+^) and lipid ROS	Fe	-	Shang et al. (2020)
miR-22 (miRNA)	Increase ROS	SIRT-1	+	Pant et al. (2017)
miR-92 (miRNA)	Increase ROS	unknown	+	Cardin et al. (2012)
miR-145 (miRNA)	Elimination of insulin-induced PKM2 and ROS elevation	PKM2	-	Li et al. (2014)
miR-222 (miRNA)	Unknown	ER (endoplasmic reticulum)	-	Dai et al. (2010)
Let-7 (miRNA)	Directly acts on the 3′-UTR of Bach1 and negatively regulates expression of this protein, and thereby up-regulates modulation of heme oxygenase 1 (HMOX1) gene expression	Heme oxygenase-1	-	Hou et al. (2012)
miR-221 (miRNA)	Unknown	ER	-	Dai et al. (2010)
miR-21 (miRNA)	Increase ROS	unknown	+	Shu et al. (2016)
miR-181 (miRNA)	Increase ROS	Unknown	+	Zhang et al. (2020)
miR-200a-3p (miRNA)	Inhibite p38/p53/miR-200 feedback loop and increased ROS	p53	+	Xiao et al. (2015)
miR-125b (miRNA)	Increase ROS	HK2	+	Li et al. (2017)
miR-26a (miRNA)	Regulate fatty acid and cholesterol homeostasis and decreasing ROS	Triglyceride, totalcholesterol, malondialdehyde	-	Ali et al. (2018)
miR-885-5p (miRNA)	Induce TIGAR (TP53-induced glycolysis and apoptosis regulator)expression through a p53-independent pathway and decreasing ROS	TIGAR	-	Zou et al. (2019)
miR-150-3p (miRNA)	Induced by ROS	/	/	Wan et al. (2017)
miR-1915-3p (miRNA)	Induced by ROS	/	/	Wan et al. (2017)
miR-34a-3p (miRNA)	Induced by ROS	/	/	Beccafico et al. (2015)
miR-34a-5p (miRNA)	Induced by ROS	/	/	Wan et al. (2017)
miR-638 (miRNA)	Induced by ROS	/	/	Wan et al. (2017)
H19 (ncRNA)	Decrease ROS	MAPK/ERK signaling pathway	-	Ding et al. (2018)
GABPB1-AS1 (lncRNA)	Downregulate the gene encoding Peroxiredoxin-5 (PRDX5) peroxidase and the eventual suppression of the cellular antioxidant capacity	/	+	Qi et al. (2019)
miR-18a (miRNA)	Downregulate the expression of Glutamate-Cysteine Ligase Subunit Catalytic (GCLC), the rate-limiting enzyme of GSH synthesis	GSH	+	Anderton et al. (2017)
miR-152 (miRNA)	Reduce GSH levels by targeting Glutathione S-transferase	GSH	+	Huang et al. (2010)
miR-503 (miRNA)	Unknown	GSH	+	Wang et al. (2014)
Neat1 (lncRNA)	Increase GST to increase GSH consumption	GST	+	Wang et al. (2018)
Metallothionein-1G (MT-1G)	Induce depletion of GSH	GSH	-	Sun et al. (2016b)
Deleted in azoospermia-associated protein 1 (DAZAP1)	Interact with the 3′UTR (untranslated region) of SLC7A11 mRNA and positively regulate its stability	SLC7A11	-	Wang et al. (2021b)
Transforming growth factor β1 (TGF-β1)	Upregulate of Smad3 inhibits SLC7A11 expression	SLC7A11	+	Kim et al. (2020)
sulfasalazine	Inhibit SLC7A11	SLC7A11	+	Song et al. (2017)
Actinomycin D	Inhibit of SLC7A11 expression by inhibition of CD133 synthesis	SLC7A11	+	Song et al. (2017)
Circ0097009 (circRNA)	Regulate of SLC7A11 expression by expression of miR-1261	SLC7A11	-	Lyu et al. (2021)
METTL14	SLC7A11 mRNA was modified at 5′UTR and degraded	SLC7A11	+	Fan et al. (2021)
transcription factors YAP/TAZ	Induce the expression of SLC7A11	SLC7A11	-	Gao et al. (2021)
IFN-γ	Down-regulate the mRNA and protein levels of SLC3A2 and SLC7A11	SLC7A11	+	Kong et al. (2021)
activating transcription factor 3 (ATF3)	Bind to the SLC7A11 promoter and repressing SLC7A11 expression in a p53-independent manner	SLC7A11	+	Wang et al. (2020)
miR-182-5p and miR-378a-3p (miRNA)	Directly bind to the 3′UTR of GPX4 and SLC7A11 mRNA, downregulation of GPX4 and SLC7A11	GPX4, SLC7A11	+	Ding et al. (2020)
LINC00618 (lncRNA)	Increase the levels of lipid ROS and iron, decreasing the expression of SLC7A11	ROS,SLC7A11	+	Wang et al. (2021c)
microRNA-17-5p (miRNA)	Activate the p38 MAPK pathway, which in turn facilitates the phosphorylation of HSPB1	HSPB1	unknown	Yang et al. (2010)
heat shock protein beta-1 (HSPB1)	Reduce iron-mediated production of lipid ROS	ROS	-	Sun et al. (2015)
protein kinase p38α (Mapk14)	Decrease the expression of HSPB1 to reduce the accumulation of intracellular ROS	HSPB1	+	Sakurai et al. (2013)
dual specificity phosphatase 1 (DUSP1)	Inhibit the phosphorylation of P38 MAPK and HSPB1	HSPB1	+	Hao et al. (2015)
Astragalus	Directly down-regulate MT1G	MT1G	+	Liu et al. (2021b)
microRNA-205 and microRNA-211-5p (miRNA)	Target the 3ʹUTR of ACSL4 inhibits ACSL4 expression at mRNA and protein levels	ACSL4	-	Cui et al. (2014); Qin et al. (2020)
Lactic acid	Produce sterol regulatory element binding protein 1 (SREBP1) and downstream stearoyl-coA desaturase-1 (SCD1) to enhance the production of iron-resistant monounsaturated fatty acids (PUFA). SCD1 acts synergistically with acyl-CoA synthase 4 (ACSL4)	ACSL4,PUFA	-	Zhao et al. (2020)
NADPH-cytochrome P450 reductase (POR) and NADH-cytochrome b5 reductase (CYB5R1)	React with iron to generate reactive hydroxyl radicals for the peroxidation of the polyunsaturated fatty acid (PUFA) chains of membrane phospholipids, thereby disrupting membrane integrity	PUFA	+	Yan et al. (2021)
DJ-1/PARK7 (cancer-associated protein)	DJ-1 depletion inhibits the transsulfuration pathway by disrupting the formation of the S-adenosyl homocysteine hydrolase tetramer and impairing its activity	homocysteine	-	Cao et al. (2020)
hydroxycarboxylic acid receptor 1 (HCAR1)/monocarboxylate transporter 1 (MCT1)	Enhance the production of anti-ferroptosis monounsaturated fatty acids	MUFA	-	Zhao et al. (2020)

### (Anti-)Oxidant Metabolism

(Anti-)oxidant Metabolism plays an important role in ferroptosis. Glutathione (GSH) metabolism and anti-oxidant capacity regulate sensitivity to ferroptosis. GSH is a tripeptide antioxidant that acts as a cofactor of Se-dependent GPX4 to reduce lipid hydroperoxides (Yant et al., 2003; Lu, 2009). Inhibition of cystine required for GSH synthesis eventually leads to depletion of intracellular GSH levels (Dixon et al., 2012; Dixon and STOCKWELL, 2014). GPX4 converts GSH between the reduced and oxidized states and converts lipid hydroperoxides to lipid alcohols. This process prevents the formation of Fe^2+^ dependent toxic lipid ROS (Labunskyy et al., 2014; Forcina and DIXON, 2019). GPX4 is the only reported enzyme that can directly reduce complex phospholipid peroxides and is the downstream target gene of NRF2 (Nuclear factor E2-related factor 2) (Forcina and DIXON, 2019; Friedmann Angeli et al., 2019). Erastin, a classical ferroptosis-inducing drug, depletes GSH and indirectly inactivates GPX4, leading to accumulation of toxic lipid ROS and subsequent lipid peroxidation (Dixon et al., 2012; Dixon and STOCKWELL, 2014), ultimately leading to ferroptosis.

At present, most studies on NRF2 in HCC involve the p62-Keap1 (Kelch-like ECH-associated protein 1)-NRF2 axis. The p62-Keap1-NRF2 signaling pathway is involved in the process of cell avoiding ferroptosis. NRF2 is a key regulator of the antioxidant response, including the expression of the Cystine/glutamate exchange system (system X^C−^) (Hassannia et al., 2019). Inhibition or knockdown of NRF2 enhances erastin- or sorafenib-induced ferroptosis in HCC *in vitro* and *in vivo* (Hassannia et al., 2019). The System X^C−^ consists of solute carrier family 7 member 11 (SLC7A11, xCT) and solute carrier family 3 member 2 (SLC3A2, 4F2hc) by disulfide bonded, which import the extracellular oxidized form of cysteine and cystine, in exchange for intracellular glutamate. SLC7A11 indirectly inactivates GPX4 by reducing cysteine uptake, thereby limiting GSH synthesis, increasing lipid ROS, and ultimately leading to ferroptosis (Sato et al., 1999; Cao and DIXON, 2016). NRF2 has antioxidant elements and is regulated by Keap1. Its gene transcription is partially under the control of ROS. Sun et al. (2016a) found p62 expression prevents NRF2 degradation by Keap1 inactivation and enhances the subsequent nuclear accumulation of NRF2. They also demonstrate that NRF2-mediated anti-ferroptosis activity depends on the induction of NADPH (Reduced Nicotinamide Adenine Dinucleotide Phosphate) quinone oxidoreductase 1 (NQO1), heme oxygenase-1(HO-1), and ferritin heavy chain-1 (FTH1).

In morphology, ferroptosis mainly occurred in cells with reduced mitochondrial size, increased bilayer membrane density, and decreased or disappeared mitochondrial crest (Dixon et al., 2012; Yang and STOCKWELL, 2008; Yagoda et al., 2007). Mitochondria are the main source of ROS. Excessive ROS can cause significant oxidative stress and lead to cell and tissue damage (Czaja et al., 2013). Gao et al. (2019)showed that ROS derived from mitochondria are involved in cysteine deprivation induced ferroptosis. Li et al. (2021) found depletes cysteine can enhance sorafenib-induced ferroptosis and lipid ROS production, and increase oxidative stress and mitochondrial ROS accumulation. And they point out that sorafenib exerts its anti-HCC function partly by targeting the mitochondrial function. Huang et al. (2021a) found the use of ZZW-115 (Nuclear protein 1 inhibitor) induced ferroptosis and subsequent mitochondrial morphological changes, including the disintegration of mitochondrial network and severe mitochondrial metabolic disorders, which were compatible with the process of ferroptosis, and this process can be complementary to TFAM (a core mitochondrial transcription factor) (Zhao, 2019).

### Iron Metabolism

Iron is a redox-active metal that can participate in the formation of free radicals and the propagation of lipid peroxidation. Elevated iron levels increase susceptibility to ferroptosis. Iron overload or excessive activity of heme oxygenase 1 (HMOX1) increases the labile iron pool (LIP) that cause ferroptosis. Excessive iron increases ROS through Fenton reaction (through reaction with hydrogen peroxide (H_2_O_2_), ferrous iron (Fe^2+^) is oxidized into trivalent iron (Fe^3+^), forming highly active hydroxyl radical) (Hassannia et al., 2019), ROS is reversely neutralized by iron (Arefieva et al., 2021). Iron metabolism mainly involves the interaction between transferrin (TF) and its receptor (TFR), the input of iron through divalent metal transporter 1 (DMT1), the storage of iron as ferritin and iron-sulfur clusters (ISC), and the output of iron through iron transporter (FPN) (Abeyawardhane and LUCAS, 2019; Wang et al., 2019a).

The protection of the p62-Keap1-NRF2 signaling pathway on ferroptosis in HCC cells also involves the regulation of Fe homeostasis. An early study showed an increase in TFR1 and a decrease in ferritin (FTL and FTH1) expression in ferroptosis sensitive cells compared with iron-resistant cells (Yang and STOCKWELL, 2008). Sun et al. (2016a) showed that it was FTH1, not FTL or TFR1, that was regulated by NRF2 in ferroptosis. FTH1 inhibited ferroptosis by storing and transporting Fe^2+^ in HCC cells. In addition, excess iron in the liver may play a role in carcinogenesis by promoting tumor growth and altering the immune system (Kowdley, 2004). It is important to note that induction of ferroptosis in the liver may have different roles in tumorigenesis and cancer therapy.

### Lipid Metabolism

Ferroptosis is iron-dependent regulatory necrosis induced by lipid peroxidation that occurs in cell membranes, a peroxidation reaction by polyunsaturated fatty acids catalyzed by the synthesis of acyl-CoA synthetase long-chain family member 4 (ACSL4) (Doll et al., 2017; Conrad and PRATT, 2019). Some polyunsaturated fatty acids (PUFAs) such as phosphatidylethanolamine (PE) and phosphatidylcholine (PC) are responsible for inducing ferroptosis by lipid peroxidation. Since *de novo* synthesis of PUFAs is strictly limited in mammals, various PUFAs are produced by the PUFAs biosynthesis pathway through the uptake of essential fatty acids from the blood and lymphatic fluid by cells. Free polyunsaturated fatty acids can be incorporated into cell membranes by various enzymes, such as ACLS4 and LPCAT3 (lysophosphatidylcholine acyltransferase 3), and lipid peroxidation can be induced by enzyme-induced and non-enzyme-induced mechanisms, resulting in ferroptosis (Lin et al., 2021). In this regard, knockdown of ACLS4, which preferably converts arachidonoyl (AA) to acylated AA, or loss of LPCAT3, which catalyzes the insertion of acylated AA into PLs (phospholipids), and make cells resistant to ferroptosis (Dixon et al., 2015; Yuan et al., 2016; Doll et al., 2017; Kagan et al., 2017). Magtanong et al. (2019) found that acyl-CoA synthetase long-chain family member 3 (ACSL3) converts monounsaturated fatty acids (MUFAs) into its acyl-CoA ester for incorporation into membrane phospholipids, thereby protecting cancer cells from ferroptosis. However, the levels of fatty acids (include MUFAs and PUFAs) in human serum are much higher than those in classical media containing fetal bovine serum (FBS), so how cells maintain the level of free fatty acid pools in cells is important to determine whether cells experience ferroptosis (Kamphorst et al., 2013; Magtanong et al., 2019).

### Energy Metabolism

Cellular energy metabolism is directly related to ferroptosis because it regulates antioxidant defense by mediating the synthesis of biological macromolecules and biological reductants such as NADPH (Zheng and CONRAD, 2020). Tumor cells typically exhibit upregulated glycolysis and PPP (pentose phosphate pathway) activity, which not only reduces ROS production by inhibiting mitochondrial respiration but also replenishes NADPH supply, thereby helps maintaining redox homeostasis to ensure cell survival. In energy metabolism, previous studies have reported that Cytochrome P450 oxidoreductase (POR) is a key mediator of ferroptosis, which promotes ferroptosis through the peroxidation of saturated phospholipids in cell membranes (Zou et al., 2020). Glucose 6-phosphate dehydrogenase (G6PD) is a key enzyme in PPP and plays a key role in NADPH production (Yang et al., 2019). G6PD may negatively regulate ferroptosis in HCC by regulating POR (Cao et al., 2021). Lu et al. (2018) pointed out that G6PD induces epithelial-mesenchymal transition (EMT) by activating the Signal Transducers and Activators of Transcription 3(STAT3) pathway, thereby promoting migration and invasion of HCC. Therefore, it can be concluded that disruption of tumor energy metabolism pathway not only changes the sensitivity of mutant tumor cells to ferroptosis, but also reduces their antioxidant defense ability to promote ferroptosis, and even affects tumor migration and invasion.

### Regulation of Ferroptosis by Non-Coding RNAs

According to length and shapes, ncRNAs are divided into various types including microRNAs (miRNAs), PIWI-interacting RNAs (piRNAs), small nuclear RNAs (snRNAs), small nucleolar RNAs (snoRNAs), long ncRNAs (lncRNAs), circular RNAs (circRNAs), transfer RNAs (tRNAs), and ribosomal RNAs (rRNAs) (Wang et al., 2019b; Alzhrani et al., 2020). MiRNAs exhibit functions by binding to the 3′-untranslated regions of target mRNAs and suppressing their expression (Majidinia et al., 2020). MiRNA can regulate ferroptosis and control cancer progression by regulating GSH, iron levels, NRF2, and ROS. LncRNAs mainly act as the regulatory factors of transcription factors in the nucleus or as miRNAs of sponges in the cytoplasm to regulate ferroptosis (Wu et al., 2020). However, there were few studies on the relationship between ferroptosis and circRNA, tRNA, rRNA, piRNA, snRNA, and snoRNA. Studies have reported that the tRNA mutations in HCC leads to decreased expression of selenoproteins, except for GPX4 and GPX1 (glutathione peroxidase 1), and introduces some weak changes in ferroptosis (Kipp et al., 2013; Becker et al., 2014; De Spirt et al., 2016). The regulation of ferroptosis found in HCC about ncRNAs in recent years was sorted out in Table 1 and Figure 1. Wider and deeper studies are needed to explore the function of ncRNAs in ferroptosis.

## Treatment of Ferroptosis in HCC

### Ferroptosis Associated With Chemotherapy Resistance in HCC

Although the treatments have become more diversified in recent years, the average life expectancy of HCC was lagged far behind those of other cancers. The result of systemic chemotherapy has been particularly disappointing, not only because of the chemotherapeutic resistance of HCC, but also the severe results of major side effects, making the treatment of advanced HCC depends on the degree of underlying liver dysfunction, the burden of malignancy, and the patient’s general profile or expectations. Treatment options for advanced HCC are limited comparing to early HCC. In this context, several therapeutic agents have been developed over the past 50 years to provide better responses and improve the average life expectancy in patients with HCC. Some common chemotherapeutic agents in HCC are summarized in Table 2. However, In two randomized clinical trials of advanced HCC patients in stage III, Sorafenib, which is a commonly used chemotherapy drug, only increased overall survival by 2.8 and 2.3 months compared to the placebo, suggested limited effect to drug-resistant HCC in advanced HCC (Llovet et al., 2008; Cheng et al., 2009). Therefore, overcome the resistance of sorafenib and find more effective new drugs has become an urgency for advanced HCC patients and postoperative adjuvant chemotherapy patients. Different regulatory strategies and delivery routes have been proposed to enhance the antitumor activity of these drugs (Kodama et al., 2008; Hung et al., 2012; Li et al., 2013; Song et al., 2013). Although some ferroptosis inducers, for example, Erastin, are very effective in killing cancer cells *in vitro*, their pharmacokinetic properties, such as solubility and metabolic stability, are not suitable for the usage *in vivo* (Yang et al., 2014). It is now believed that sorafenib can induce a new type of regulated cell death-ferroptosis (Louandre et al., 2013), distinct from apoptosis, necrosis, and autophagy (Dixon et al., 2012), not only sorafenib, Guo et al. (2018) killed a variety of tumor cells with cisplatin, which can simultaneously cause apoptosis and ferroptosis. Wu et al. (2018) found that some ncRNAs affect the sensitivity of 5-Fu-resistant cells by regulating some key steps of ferroptosis.

**TABLE 2:** Common chemotherapeutic agents in HCC.

Chemotherapeutic agent	Mode of action	References
Sorafenib	Tyrosine-kinase inhibitor	Shaaban et al. (2014)
5-Flurouracil	Inhibition of thymidylate synthase	Longley et al. (2003)
Cisplatin	DNA damage	Shaaban et al. (2014)
Gemcitabine	Nucleotide analogue mis-incorporated into DNA	Heinemann et al. (1988); Mini et al. (2006)
Capecitabine	Inhibition of DNA synthesis	Walko and LINDLEY (2005)
Doxorubicin	Generation of free radicals and the intercalation into DNA	Gewirtz, (1999)
Epirubicin	Inhibitor of DNA topoisomerase II	Shaaban et al. (2014)
Lenvatinib	An inhibitor of VEGF receptors 1–3, FGF receptors 1–4, PDGF receptor α, RET, and KIT	Kudo et al. (2018)

In recent years, the adjustment of HCC-related chemotherapy resistance is shown in Table 3.

**TABLE 3:** The adjustment of hepatocellular cancer-related chemotherapy resistance.

Gene/Axis/Compound/Drug	Mechanism	Target	Influence to drug resistance	References
Aspirin	Silences of ACSL4 and induction of GADD45B expression	ACSL4	synergized with sorafenib	Xia et al. (2017)
GSTZ1	Inhibit NRF2/GPX4 axis	GPX4	synergized with sorafenib	Wang et al. (2021a)
QSOX1	Inhibit NRF2	NRF2	synergized with sorafenib	Wang et al. (2021a)
MT-1G	Knockout of MT-1G increases glutathione consumption and lipid peroxidation	MT-1G	synergized with sorafenib	Sun et al. (2016b)
Malic enzymes (MEs)	Produce NADPH and neutralizes ROS	NRF2	synergized with sorafenib	Lee et al. (2021)
Astragalus	Directly down-regulate MT-1G	MT-1G	synergized with sorafenib	Liu et al. (2021b)
Secreted protein acidic and rich in cysteine (SPARC)	LDH release and ROS accumulation	ROS	synergized with sorafenib	Hua et al. (2021)
Artesunate	Degradation of ferritin, lipid peroxidation	lysosomal	synergized with sorafenib	Li et al. (2021c)
disulfiram/copper	Inhibit NRF2 and MAPK kinase signaling pathways	NRF2	synergized with sorafenib	Ren et al. (20211021)
Haloperidol	Antagonize sigma receptor 1	S1R	synergized with sorafenib	Bai et al. (2017)
CISD2	Excessive iron ion accumulation	FE	synergized with sorafenib	Li et al. (2021b)
Transcription factors YAP/TAZ	Induce SLC7A11 expression	SLC7A11	Antagonism with sorafenib	Gao et al. (2021)
Apoptosis-inducing factor mitochondria-associated 2 (AIFM2)	Activation of membrane repair mechanisms that regulate membrane germination and fission	unknown	Antagonism with sorafenib	Dai et al. (2020)
Sigma-1 receptor (S1R)	Inhibit the accumulation of ROS	NRF2	Antagonism with sorafenib	Bai et al. (2019)
DAZAP1	Interact with the 3′UTR (untranslated region) of SLC7A11 mRNA and positively regulated its stability	SLC7A11	Antagonism with sorafenib	Wang et al. (2021b)
Sulfasalazine	Inhibit SLC7A11	SLC7A11	associated with drug resistance of cisplatin, doxorubicin and sorafenib	Song et al. (2017)
miR-340 (miRNA)	Targetes NRF2	NRF2	synergized with cisplatin	Shi et al. (2014)
Apigenin	Inhibit Mir-101/Nrf2 pathway	NRF2	synergized with doxorubicin	Gao et al. (2017)
KRAL (lncRNA)	Induce Keap1 to regulation NRF2	NRF2	synergized with 5-Fluorouracil (5-FU)	Wu et al. (2018)
miR-144 (miRNA)	Targete NRF2	NRF2	synergized with 5-Fluorouracil (5-FU)	Zhou et al. (2016)
ATP-binding cassette C5 (ABCC5)	Stabilize SLC7A11 protein to increase intracellular GSH and attenuate lipid peroxidation accumulation	SLC7A11	Antagonism with sorafenib	Huang et al. (2021b)
Ungeremine	Increase ROS production	ROS	related	Mbaveng et al. (2019)
XCanthine oxidoreductase (XOR)	NRF2 degradation	NRF2	related	Sun et al. (2020)

### Ferroptosis Associated With Radiotherapy Tolerance in HCC

Radiation therapy is an important non-surgical treatment for cancer, but the clinical problems such as low efficacy and severe side effects remained unsolved. Gene therapy can synergistically increase the effect of radiation therapy through its antitumor mechanisms, which may reduce the dose. Radiotherapy induces ferroptosis by down-regulation of SLC7A11 and up-regulation of ACSL4, resulting in GSH production, increasing lipid synthesis, and subsequent oxidative damage (Lang et al., 2019; Lei et al., 2020). Studies have found that collectrin (CLTRN), as a target of radiation, is regulated by NRF1 (nuclear respiratory factor 1)/RAN (RAS oncogene family)/DLD (dihydrolipoamide dehydrogenase) protein complex and enhances the radiosensitivity of HCC cells through ferroptosis (Yuan et al., 2021). A combination of gene therapy and radiation therapy is one way forward, allowing the radiation doses to be reduced and the side effects to be reduced. It is worth considering whether the application of iron death inhibitors to non-tumor cells can increase their radiation tolerance to reduce the adverse effects of radiotherapy.

### Ferroptosis Associated With Emerging Therapies in HCC

The use of nano drugs to induce ferroptosis will become a new anticancer strategy (Shen et al., 2018). More and more anticancer nano drugs have been approved by FDA, and the development of drugs with higher efficacy and safety will become an emerging road for future cancer treatment (Bobo et al., 2016). Tang et al. (2019) synthesized manganese-doped mesoporous silica nanoparticles (MMSNs) from manganese and silica. This reaction resulted in the inactivation of GPX4 and the increase of intracellular lipid peroxides through the consumption of intracellular GSH induced by the degradation of MMSNs. Ou et al. (2017) used natural omega-3 fatty acid docosahexaenoic acid (LDL-DHA) reconstructed into Low-density lipoprotein nanoparticles to selectively kill HCC cells. LDL-DHA induces ferroptosis by increasing tissue lipid hydroperoxide levels and inhibition of GPX4 expression. Tian et al. (2022) reported a novel cascade copper-based metal-organic framework (MOF) therapeutic nanocatalyst using HKUST-1 (a kind of metal organic framework) combining meloxicam (Mel), a cyclooxygenase-2 (COX-2) inhibitor, and sorafenib (Sol). Down-regulation of COX-2 induces PINK1/Parkin-mediated mitochondrial autophagy, chemodynamic Therapy (CDT) -mediated cytotoxic ROS, accumulated lipid peroxides (LPO) and Sol through inhibition system X^C−^, the three interacted to activate ferroptosis and increase the sensitivity of HCC cells to chemotherapy. Liu et al. (2021a) constructed mil-101 (Fe) nanoparticles (NPs) loaded with sorafenib and iRGD (iRGD peptide (amino acid sequence: CRGDK/RGPD/EC) [MIL-101 (Fe) @ SOR], which co-administration significantly promoted the development of ferroptosis. Ma et al. (2017) enhanced the sensitivity of cancer cells to cisplatin by loading cisplatin prodrug onto iron oxide nanoparticles to increase ROS production. Du et al. (2021) designed an exosome with three parts, including surface functionalization of CD47, membrane loading of ferroptosis inducer Er (Erastin), and core of photosensitizer RB (Rose Bengal), and demonstrated potent antitumor therapeutic effects with surprisingly low toxicity.

## Discussion

In this review, we summarize recent advances in potential regulators of ferroptosis in HCC and look into the ways ferroptosis can be used to create new therapies in the future. We demonstrate multiple advances in the drug resistance assessments in HCC treatment, the use of multiple genes or compounds to sensitize sorafenib, and the treatment of ferroptosis in HCC in some emerging areas, Nanoparticles such as MMSNs and LDL-DHA prepared in the tumor microenvironment and engineered exosomes with ferroptosis inducers are utilized to induce ferroptosis to bring better prognosis for patients.

The combination of ferroptosis with other therapies, such as immunotherapies, is also promising. Recently, it has been reported that anti-PD-L1 (programmed cell death-Ligand 1) immune checkpoint blockade can induce cancer cell ferroptosis responses by down-regulating SLC7A11 expression in cancer cells as a result of IFN-γ (Interferon γ) secreted by CD8^+^ T cells (Wang et al., 2019c). Therefore, we believe that therapeutic expansion in ferroptosis may realize effective treatment for patients with advanced HCC.

There are still some issues to be resolved: Although lipid peroxidation is an important factor affecting ferroptosis, what is the actual mechanism of ferroptosis downstream of phospholipid peroxidation? There are many mechanisms of ferroptosis, and many metabolic factors affect the death of tumor cells, the formation of drug resistance, and the avoidance of immune-induced metastasis. It is still unknown that which metabolic factor plays a more important decisive role. *In vivo* pharmacokinetics of some ferroptosis inducers are still not suitable for *in vivo* usage, especially how ferroptosis drugs work in liver-specific biotransformation in the treatment of HCC. The fatty acid pool of cells affects the progress of ferroptosis in cells, how to use the change of fatty acid in the blood to determine the progress of ferroptosis in cells? and how to create a fatty acid microenvironment that is conducive to killing tumor cells in the liver?
